# In the June 2016 issue

**DOI:** 10.1590/S1980-5764-2016DN1002001

**Published:** 2016

**Authors:** Ricardo Nitrini

In this issue we are publishing three reviews, six original papers, four case reports and one case in the section ´Neurology through clinical cases´. 

Engedal and Laks reviewed the common goals of European dementia plans for improving dementia care. Knowledge of the successful achievements and the identification of barriers to implementation in other countries should help the design of a Brazilian plan for dementia. 

Schilling et al. highlighted the most important contributions of PET molecular imaging for early diagnosis of Alzheimer's disease (AD), gaining a better understanding of the pathophysiological process and monitoring disease progression. 

Ribas el al. reviewed historical aspects of The Learning Curve, a training technique based on the relationship between brain electrical activity and cognitive/behavioral states. 

Ribeiro and Busatto systematically reviewed voxel-based morphometry (VBM) studies addressing the frontal lobes in AD. Most studies reported reduced gray matter volume in the frontal lobes while a few studies also reported this finding in very mild AD. 

Holderbaum et al. investigated the semantic priming effect (SPE) after left hemispheric stroke looking for dissociation in performance on the lexical decision task. Three groups of patients were identified according to the SPE. 

Simon et al. translated the Multifactorial Memory Questionnaire (MMQ) to Brazilian Portuguese and applied the instrument to elderly subjects. This version showed good psychometric properties and seems promising for clinical and research purposes. 

Batini et al. evaluated the effects of a narrative training program on performance in memory tests. In this pilot study, significant improvements in scores on immediate and delayed memory tests were found. 

Batistela et al. investigated the effect of methylphenidate as a cognitive enhancer in 36 healthy young subjects. No difference was observed in performance on tests of attention and memory after daily doses of 10 to 40 mg. 

Dalpai et al. evaluated the association between characteristics of professional caregivers and the strategy for managing demented patients. A positive correlation was found between caregiver burden and criticism, a strategy based on authoritative attitudes by the caregiver toward the elderly. 

Guedes et al. reported a case with psychosis, punding, and pathological gambling in a patient with Parkinson disease to emphasize the importance of dopamine replacement therapy in the genesis of these behavior disturbances.

Assed et al. reported a preliminary study of cognitive training in an 80-year-old man with memory complaints, using a computer program known as Neurotracker. After 32 training sessions, there were improvements in performance on memory tests and in memory complaints. 

Andrade et al. presented a preliminary report of the treatment of a case of Alzheimer's disease with transcranial direct current stimulation associated with acetylcholinesterase inhibitor (donepezil) and cognitive training. Improvements were observed on several cognitive tests pointing to the need for further studies. 

Pereira et al. reported a genetically-proven case of pantothenate kinase-associated neurodegeneration (PKAN), a neurodegenerative disorder with brain iron accumulation. The characteristic clinical and MRI presentations with the eye of tiger sign were shown. 

Vieira et al. reported a case of progressive multifocal leukoencephalopathy in a patient with an indolent non-Hodgkin lymphoma describing the MRI characteristics of the disease at presentation and the evolution of MRI findings.

Ricardo Nitrini Editor-in-Chief

